# Possible Anandamide and Palmitoylethanolamide involvement in human stroke

**DOI:** 10.1186/1476-511X-9-47

**Published:** 2010-05-14

**Authors:** Marcello Naccarato, Daniela Pizzuti, Stefania Petrosino, Marco Simonetto, Laura Ferigo, Fabio Chiodo Grandi, Gilberto Pizzolato, Vincenzo Di Marzo

**Affiliations:** 1 Department of Medical, Technological and Translational Sciences, Neurology Unit, University of Trieste, Strada di Fiume 447, 34100 Trieste (TS), Italy; 2 Department of Surgical and Gastroenterological Sciences, University of Padova, Via Giustiniani 5, Padova (PD), Italy; 3 Endocannabinoid Research Group, Institute of Biomolecular Chemistry, National Research Council, Via Campi Flegrei 34, Pozzuoli (NA), Italy

## Abstract

**Background:**

Endocannabinoids (eCBs) are ubiquitous lipid mediators that act on specific (CB1, CB2) and non-specific (TRPV1, PPAR) receptors. Despite many experimental animal studies proved eCB involvement in the pathogenesis of stroke, such evidence is still lacking in human patients. Our aim was to determine eCB peripheral levels in acute stroke patients and evaluate their relationship with clinical disability and stroke volume.

**Methods:**

A cohort of ten patients with a first acute (within six hours since symptoms onset) ischemic stroke and a group of eight age- and sex-matched normal subjects were included. Groups were also matched for metabolic profile. All subjects underwent a blood sample collection for anandamide (AEA), 2-arachidonoylglycerol (2-AG) and palmitoylethanolamide (PEA) measurement; blood sampling was repeated in patients on admission (T0), at 6 (T1) and 18 hours (T2) thereafter. Patients neurological impairment was assessed using NIHSS and Fugl-Meyer Scale arm subitem (FMSa); stroke volume was determined on 48 h follow-up brain CT scans. Blood samples were analyzed by liquid chromatography-atmospheric pressure chemical ionization-mass spectrometry.

**Results:**

1)T0 AEA levels were significantly higher in stroke patients compared to controls. 2)A significant inverse correlation between T0 AEA levels and FMSa score was found. Moreover a positive correlation between T0 AEA levels and stroke volume were found in stroke patients. T0 PEA levels in stroke patients were not significantly different from the control group, but showed a significant correlation with the NIHSS scores. T0 2-AG levels were lower in stroke patients compared to controls, but such difference did not reach the significance threshold.

**Conclusions:**

This is the first demonstration of elevated peripheral AEA levels in acute stroke patients. In agreement with previous murine studies, we found a significant relationship between AEA or PEA levels and neurological involvement, such that the greater the neurological impairment, the higher were these levels.

## Background

During the last decade numerous studies have addressed the role of the endocannabinoid (eCB) system in different pathological conditions. Endocannabinoids (eCBs), e.g. anandamide (AEA) and 2-arachidonoylglycerol (2-AG), are lipid mediators synthesized "on demand" that inhibit neurotransmitter (glutamate and GABA) release and modulate neuroinflammation by activating specific CB_1 _(highly expressed in the CNS, where they mediate the psychotropic effects of Δ^9^-tetrahydrocannabinol) and CB_2 _(expressed by immune cells, including brain resident microglial cells) receptors, respectively. Cannabinoid receptor-inactive eCB-related molecules, e.g. palmitoylethanolamide (PEA), also exert neuroprotective effects[1-3], presumably by preventing mast cell degranulation [4], and directly activating peroxisome proliferator-activated receptor (PPAR)-α [5], or by enhancing the effects of AEA on cannabinoid receptors, transient receptor potential vanilloid type-1 (TRPV1) channels and PPAR-γ receptors [6].

Previous murine and cell culture studies on stroke and hypoxia postulated a neuroprotective role of eCBs, given their ability to decrease NMDA-mediated toxicity in vascular penumbra through a CB_1_-mediated mechanism [7]. Increases of AEA content, of the AEA biosynthetic precursors (e.g. N-acyl phosphatidylethanolamines), and of CB_1 _receptors expression in ischemic brain regions of murine stroke models have been described [8-11]. CB_1 _knockout mice develop larger stroke volumes than wild-type animals, with consequent increased post-stroke disability and mortality [12]. In addition, CB_1 _agonist administration was associated with a decrease of infarct volume and with an improvement of clinical symptoms in stroke-treated mice [13]. Interestingly, there is evidence that also low-doses of CB_1 _receptor antagonists, such as rimonabant, reduce infarct volume in stroke models [9,10,14,15], possibly by enhancing TRPV1-mediated actions [14]. It has been also suggested that part of the neuroprotective effects of CB_1 _receptor agonists in stroke is due to their capability of lowering body temperature, and that CB_1_, as opposed to CB_2_, receptors might otherwise play a counterprotective role in cerebral ischemia [15].

Indeed, also CB_2 _receptors have been implicated in the pathogenesis of stroke. Since such receptors are particularly expressed on activated microglia and peripheral immune cells (mastcells, macrophages and lymphocytes), they may act by modulating the inflammatory response to stroke [16], which is triggered 24-48 hours after symptoms onset and is mainly responsible for the delayed neuronal death [17]. Indeed CB_2 _agonists administration was associated with a reduction of infarct volume and neurological impairment in murine models of stroke and cerebral ischemia [15,18]. However, such evidence is still lacking in humans, despite the fact that a single case report described an increase of AEA and PEA content in the ischemic hemisphere of a stroke patient [19].

Aim of this study was to evaluate the possible involvement of the eCB system in stroke patients, by measuring plasma AEA, 2-AG, and PEA levels in the acute phase of the disease and by correlating eCB and PEA plasmatic levels with measures of neurological impairment and volume of the ischemic brain tissue.

## Methods

### Subjects

10 patients (Group A; M/F = 5/5; mean age 70 ± 13 years) affected by a first ischemic stroke involving the Middle Cerebral Artery (MCA) territory with at least arm impairment and symptoms onset ≤ 6 hours before admission, and 8 age-matched healthy volunteers (Group B; M/F = 4/4; mean age 70 ± 12 years) were enrolled. Patients with other neuropsychiatric diseases, substance and/or alcohol abuse, or systemic inflammatory diseases were excluded. The study was approved by the local institutional ethical committee and each participant (or relative, in case of aphasia/impaired consciousness) gave written informed consent in accordance with the Declaration of Helsinki. On admission (T0), patients underwent CT brain scanning, evaluation of neurological impairment with the NIHSS [20] and the Fugl-Meyer arm subitem scale (FMSa) [21], calculation of the Body Mass Index (BMI), blood sampling for routine laboratory analysis (Cholesterol, Triglycerides, Fasting Blood Glucose) and for eCB measurements. Blood sampling for eCB determination was repeated at 6 hours (T1) and 18 hours (T2) after admission. A follow-up brain CT scan was performed 48 hours after admission.

Control subjects (Group B), after written informed consent, underwent blood sampling for eCB and laboratory analysis; their BMI was also calculated.

### Endocannabinoid determination

Blood was collected in EDTA and immediately centrifuged at 400 g for 30 min at room temperature. Extraction, purification of AEA, 2-AG and PEA, and their quantification by isotope-dilution liquid chromatography-atmospheric pressure chemical ionization-mass spectrometric analysis were performed as previously described [22].

### Brain CT analysis

**Figure 1:**
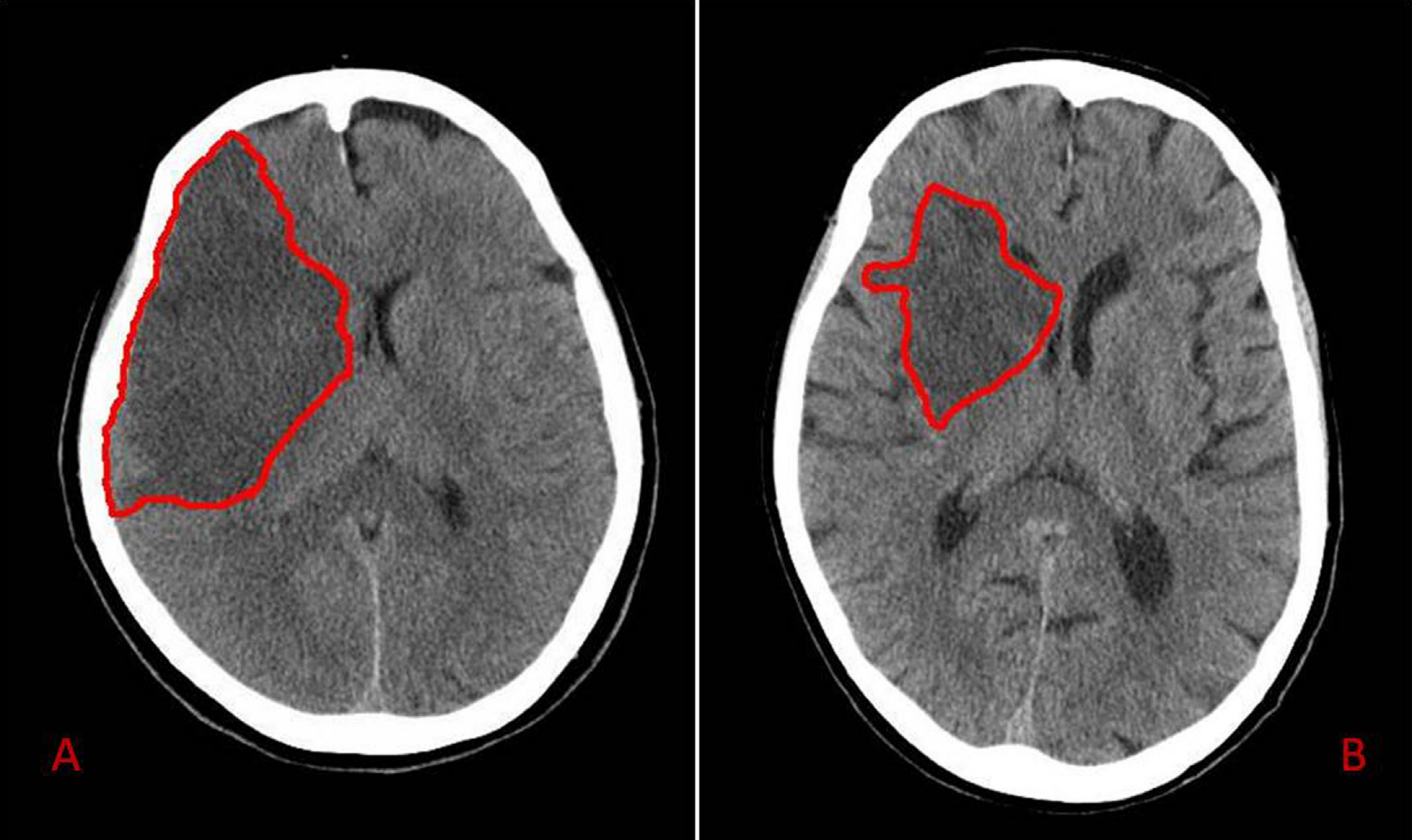
**Follow-up brain CT scans of two stroke patients**. Two examples of CT scans of two stroke patients at 48 hours since admission. The red outline circumscribes the ischemic area. The patient in panel A had a total occlusion of right middle cerebral artery, presenting with left hemiparesis and hemianopia (Female; Age = 80 yrs.; T0 AEA = 7.31 pmol/lipid mg; T0 NIHSS = 17; T0 aFMS score = 12). The patient in panel B was admitted for a left hemiparesis due to a partial occlusion in right middle cerebral artery territory (Female, Age = 82 yrs.; T0 AEA = 3.93 pmol/lipid mg; T0 NIHSS = 10; T0 aFMS score = 18).

Twenty-eight contiguous 5 mm-thick axial slices, covering the whole brain, were obtained for each patient with a multislice TOSHIBA AQUILION scanner, with 120 kV, 250 mA and 2 s acquisition time (Toshiba Medical Systems). Thereafter, using Analyze 4.5 (Biomedical Imaging Resource, Mayo University) and a semi-automated routine, two blinded examiners (FCG and GP) calculated the total lesion volume in mL, as the sum of the stroke area in each slice multiplied by slice thickness (Fig. 1).

### Statistical analysis

Using the SPSS 13.0 statistical software package (SPSS Inc.), inter-group differences were evaluated with the Mann-Whitney U test, while the Spearman rho test was used to evaluate correlations between eCBs plasma levels and the clinical and radiological data.

## Results

Groups were comparable with regard to age, gender, BMI, and laboratory findings (Tab. 1). Mean AEA, PEA, and 2-AG blood levels in the control group (Group B) were similar to previously reported values [22-25] and correlated with total cholesterolemia and BMI.

**Table 1:** Demographic and laboratory data of stroke patients and control subjects.

	Group A	Group B	p
Sex *(M/F)*	5/5	4/4	*n.s.*
Age yrs. *(mean ± SD)*	70 ± 13	70 ± 12	*n.s.*
NIHSS score *(median ± SD; range)*	22 ± 10; 3-29	-	-
Fugl-Meyer Scale-arm score *(median ± SD; range)*	11.50 ± 9; 8-44	-	-
CT ischemic area Volume *(mL; mean ± SD)*	76.10 ± 43.46	-	-
Body Mass Index *(mean ± SD)*	25.20 ± 2.74	24.63 ± 2.39	*n.s.*
Cholesterol *(mmol/L - mean ± SD)*	11.69 ± 2.31	11.64 ± 2.04	*n.s.*
Triglycerides *(mmol/L - mean ± SD)*	6.97 ± 2.33	6.82 ± 1.00	*n.s.*
Blood Glucose (*mmol/L - mean ± SD)*	5.61 ± 0.71	5.77 ± 0.74	*n.s.*
AEA T0 *(pmol/lipid mg - mean ± SD)*	3.42 ± 2.71	1.81 ± 1.53	0.026
AEA T1 *(pmol/lipid mg - mean ± SD)*	2.87 ± 2.34	-	*n.s.*
AEA T2 *(pmol/lipid mg - mean ± SD)*	3.11 ± 2.72	-	*n.s.*
PEA TO *(pmol/lipid mg - mean ± SD)*	2.47 ± 0.96	2.05 ± 0.31	*n.s.*
PEA T1 *(pmol/lipid mg - mean ± SD)*	2.28 ± 1.01	-	*n.s.*
PEA T2 *(pmol/lipid mg - mean ± SD)*	2.17 ± 0.67	-	*n.s.*
2-AG T0 *(pmol/lipid mg - mean ± SD)*	3.42 ± 7.22	6.80 ± 9.50	*n.s.*
2-AG T1 *(pmol/lipid mg - mean ± SD)*	4.65 ± 4.98	-	*n.s.*
2-AG T2 *(pmol/lipid mg - mean ± SD)*	3.29 ± 3.10	-	*n.s.*

Demographic, clinical, radiological data and endocannabinoid or PEA plasma levels in stroke patients (Group A, at T0, T1, and T2 time-points) and control subjects (Group B). p = p values (Mann-Whitney U test); *n.s*. = not significant.

**Figure 2:**
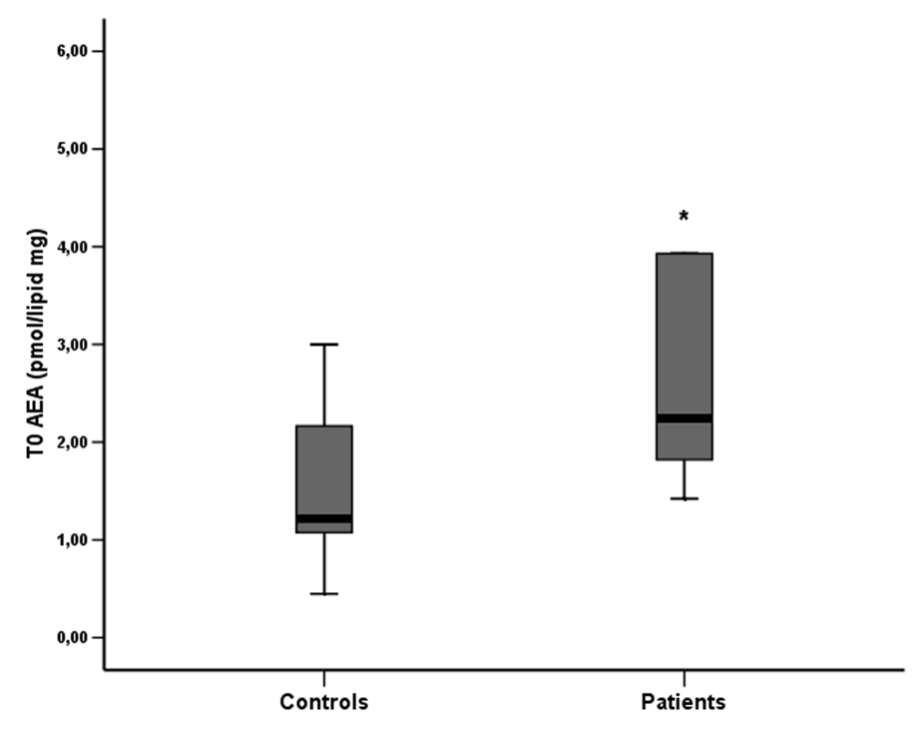
**Box-plot of plasma AEA levels in control and stroke patient groups (at T0)**. * At T0, mean plasma AEA levels were significantly higher in the stroke patients group (p < 0.05, Mann-Whitney U test).

**Figure 3:**
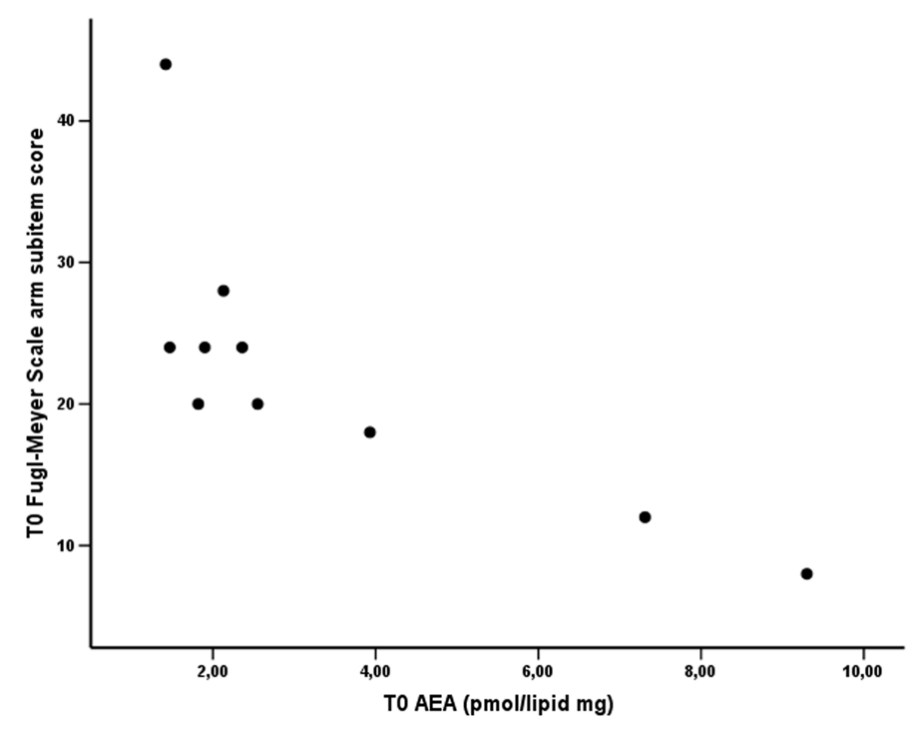
**Scatterplot of plasma AEA levels versus aFMS score in stroke patients at T0**. Significant inverse correlation between AEA levels and aFMS score (Spearman rho = -0.819, p = 0.004).

At T0, AEA plasma levels in the stroke patient group were significantly higher than in the control group (Tab. 1 and Fig. 2; Mann-Whitney U test: p < 0.05). In addition, AEA levels at T0 showed a significant inverse relationship with the FMSa scores (Fig 3; Spearman rho = -0.819, p = 0.004), so that patients with greater neurological impairment (lower FMSa score) had higher AEA levels. There was also a positive correlation between T0 plasma AEA levels and the volume of the ischemic brain region on CT scans (Spearman rho = 0.667, p < 0.05).

**Figure 4:**
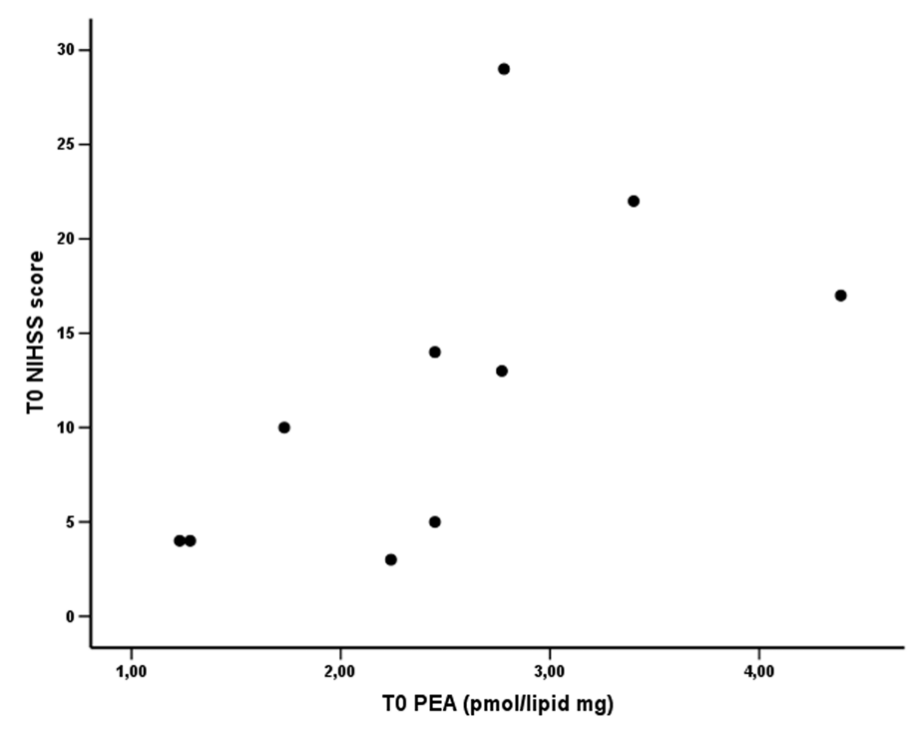
**Scatterplot of plasma PEA levels versus NIHSS score in stroke patients at T0**. Significant direct correlation between PEA levels and NIHSS score (Spearman rho = 0.823, p = 0.003).

No significant differences between groups in PEA plasmatic levels were observed. Nevertheless, there was a significant correlation between PEA levels at T0 and the NIHSS scores (Fig. 4; Spearman rho = 0.823, p = 0.003), such that the greater the neurological impairment, the higher the PEA levels.

Plasma 2-AG levels were lower in stroke patients, but group differences did not reach statistical significance.

No significant differences in AEA or PEA content were observed at later time-points (T1 and T2) between groups (Tab. 1).

## Discussion

Previous studies showed an involvement of the eCB system in various neurological conditions [26]. To our knowledge, this is the first study addressing the potential involvement of eCBs and a related mediator in acute stroke in a cohort of human patients.

Since eCBs have been proposed to act as local mediators and not as circulating hormones, the physiopathological consequences in stroke of the observed alterations in eCB plasma levels cannot be easily inferred. Nevertheless, there are several reports of changes in plasma endocannabinoid levels in patients affected by various neurological and neuropsychiatric conditions, including multiple sclerosis [27], Huntington's chorea [28], migraine [29,30], schizophrenia [22], depression [31] and anorexia nervosa [23]. Therefore, although one would expect that such conditions alter endocannabinoid signalling mostly in the brain, there is clearly a "spill-over" effect to the peripheral circulation, the extent of which often reflects, as in the present case, the severity of CNS pathology. Accordingly, endocannabinoid blood levels usually are 1-2 orders of magnitude lower than brain levels. Interestingly, a recent review by Maccarrone's group emphasized the relationship between brain diseases and endocannabinoid peripheral dysregulation, showing that peripheral eCBs could be a marker of CNS pathologies [32].

Peripheral AEA levels at T0 were significantly increased in stroke patients and showed a positive correlation with neurological disability and stroke volume. Likewise, plasma PEA levels showed a significant correlation with neurological disability. There was also a trend to reduced 2-AG levels in stroke patients. On the other hand, AEA and PEA levels at later time-points were not different between groups. These observations parallel the findings of a single case study in a patient with hemispheric stroke in which AEA and PEA contents were precociously increased in microdyalisates of tissue surrounding the ischemic lesion [19]. They also parallel several studies showing early increases of AEA and PEA, but reduced 2-AG, in ischemic brain regions in animal models of global and focal cerebral ischemia [33]. Interestingly, Maccarrone's group found an increase of CSF and plasmatic AEA, but not 2-AG, content in multiple sclerosis patients during relapses [27]. In addition, the same group recently demonstrated that an increase of striatal AEA levels may result in a decrease of 2-AG biosynthesis, thus suggesting an inverse relationship between AEA and 2-AG levels, under certain conditions [34].

The overall effect of activation of the eCB system in response to ischemia is not yet fully elucidated. In our study, plasma AEA and PEA contents in stroke patients correlated with neurological disability, but this relationship does not *per se *imply a potential neuroprotective effect. Recently, Schomacher reported that i.p. administration of AEA or PEA in rats 30 min after transient MCA occlusion significantly reduced the size of the infarcted tissue [3]. Since AEA acts as an endogenous agonist for CB_1 _and CB_2 _receptors, its neuroprotective effect may be mediated by inhibition of citotoxic glutamate release and by reducing the probability of the opening of voltage-operated calcium channels through CB_1_-mediated mechanisms. Indeed, CB_1_-knockout mice develop larger stroke volumes than wild-type animals [12]. CB_2 _mediated effects have also been implicated in stroke and in this case the eCBs may act by modulating the inflammatory response that contributes to the delayed neuronal death [33]. Such response might also be reduced by elevation of PEA levels, since PEA plays anti-inflammatory and neuroprotective effects via several potential mechanisms [3,35].

## Conclusions

Our findings in stroke patients show an early increase in plasma AEA content which correlates with neurological disability and infarct volume. Our study extends previous observations from the case report by Schäbitz and colleagues [19], showing a relationship between plasma AEA content and neurological disability. However, our present findings do not allow to determine, for instance, whether the increase in AEA levels is a consequence of stroke and exerts a possible protective effect, or whether it is part of the mechanisms leading to neurological damage during stroke. Therefore, further studies are warranted to address the therapeutic potential of eCB system modulation in stroke patients, as already explored in several (pre)clinical studies on other neurological diseases, such as multiple sclerosis, Alzheimer's disease, and brain trauma [26,36]. Finally, the role and mechanism of action of PEA in neuroprotection against cerebral ischemia also needs to be more deeply investigated.

## Competing interests

The authors declare that they have no competing interests.

## Authors' contributions

MN was involved in experimental planning, clinical data collection, data analysis and manuscript writing. DP was involved in experimental planning and endocannabinoid determination. SP performed endocannabinoid determination and contributed to data analysis. MS and LF were involved in clinical evaluation, clinical data collection and manuscript editing. FCG performed neuroimaging data analysis, contributed to data collection and manuscript editing. GP contributed to experimental planning, neuroimaging data analysis and manuscript editing. VDM contributed to experimental planning, data analysis and manuscript writing.

All authors read and approved the final manuscript.
